# Physical fitness in professional padel: performance differences according to players’ gender and playing side position

**DOI:** 10.5114/biolsport.2025.151647

**Published:** 2025-05-29

**Authors:** Francisco Pradas, Diego Muñoz, Alejandro Garcia-Gimenez, Alejandro Moreno-Azze, Bernardino J Sánchez-Alcaraz, Alejandro Sánchez-Pay

**Affiliations:** 1 Faculty of Health and Sports Sciences. ENFYRED research group, University of Zaragoza, Spain; 2 Faculty of Sports Sciences. University of Extremadura, Spain; 3 Faculty of Education. ENFYRED research group, University of Zaragoza, Spain; 4 Performance Analysis and Biomechanics in Sport, Faculty of Sport Sciences, University of Murcia, Spain

**Keywords:** Racket sports, Physical performance, Gender, Playing position, Antropometric values

## Abstract

The aim of this study was to analyse the differences in physical fitness between professional players according to their playing position (left or right side) and gender. Thirty players, 15 men and 15 women, were distributed in two groups: players playing on the right side (men n = 7; women n = 7) and the players playing on the left side (men n = 8; women n = 8). All players were assessed using a battery of anthropometric and physical performance tests, including measurements of body composition, maximal strength, aerobic capacity, speed and agility. The results showed significant differences in parameters such as weight, height and maximum strength according to playing position in male padel. Players on the left side showed higher muscle mass and strength values (one repetition maximum) in specific exercises (squat and leg curl), while those on the right side showed better results in vertical jump tests (countermovement jump and squat jump). In female players, moderate differences were observed in VO_2max_ and hand grip strength (dominant and no dominant hand), with higher values in left-side players. These results highlighted specific physical demands associated with the technical-tactical functions of each playing position. These findings provide key information to design specific training programs to optimize performance according to the player’s role on the court.

## INTRODUCTION

Physical and anthropometric testing allows coaches to identify the most relevant factors on game performance [1, 2]. In recent years, sports-science and coaching staffs are investing efforts in designing specific physical testing protocols to ensure the best possible preparation but also for the development of tracking players progress and young talent selection procedures [3, 4]. In padel, some research has implemented testing batteries combining general and specific evaluations for strength, agility, power, speed, endurance, hand-eye coordination or anthropometric measures in professional and younger players [2, 5, 6].

These field-based tests and anthropometric evaluations are gaining interest given their practical applicability, replicability, and affordability [7]. The optimal interpretation of these data is subsequently used in short- and long-terms requirements to detect injury risks, compare the players’ progress, create individual profiles and design more efficient physical training programs [8]. Despite its usefulness, fitness testing in modern padel has yet to be fully elucidated.

Padel is a racket sport that has become very popular in recent years. It currently boasts representation in over 80 countries through the International Padel Federation, increasing the number of participants in several countries [9]. Although its system of play and rules are based on other racket sports, such as tennis, padel has certain differences in comparison with it [10]. One of the differences is related to the playing court, which is characterised by dimensions of 20 m × 10 m, divided by a net, where it is surrounded by walls on the sides and the back of the court, where the ball can bounce [11, 12]. On the other hand, padel is a sport played in pairs, which influences the demands of the game, as well as the behaviour of the players, with collaborative aspects predominating [13].

The technical-tactical knowledge of the sport, the physical demands and the psychological skills are some of the components that the athlete must manage for the practice of a complex sport such as padel [13]. Regarding physical demands, padel is a predominantly aerobic sport, in which different technical stroke actions must be developed, combined with different accelerations, decelerations and changes of direction, developed intermittently in a short period of time [5, 14–17]. Recent studies highlighted the importance of understanding the dynamics of high-intensity rallies and their impact on a player’s performance, due to the variability of the technical-tactical actions [15]. These actions mentioned above require the involvement of different anaerobic performance capacities, such as speed, agility and power, where concentric and eccentric muscle actions predominate in stretch-shortening cycles [18]. These physical and technical-tactical actions at high intensities, with a high demand on anaerobic capacities, are combined with aerobic activity derived from long recovery periods between points [19, 20].

In recent years, padel research has gained significant relevance, focusing on aspects such as temporal structure, external load, game actions, and the score or players’ response to it [21]. In addition, numerous authors have further investigated this sport by carrying out various systematic reviews analysing predominant game actions, players’ movements and time structure [22]; physical, physiological and biomechanical variables [21]; and physical demands, physical level, injury risks and rehabilitation strategies [4, 23]. Research in these areas provides fundamental data that can assist coaches and strength and conditioning trainers in designing tailored programs to improve players’ performance.

Considering padel players’ fitness, body composition, static balance, upper and lower body muscular strength, flexibility, aerobic endurance and cardiovascular fitness, agility, change of direction and speed are some of the previously analysed variables, comparing them according to sex or competitive level [2, 5, 24–27]. However, there is scarce information about these differences depending on game side. Some research has shown that left-side players exhibit different movement patterns and psychological responses than right-side players, which may influence their tactical decisions and performance during matches [28]. Other studies have analysed the differences in strokes made during a match depending on the side of play [29, 30], highlighting differences in the type and number of shots according to player’s side of play, which could indicate different physical demands between them. Recent findings reveal that left-side players exhibit greater physical demands compared to right-side players. Specifically, left-side players perform more accelerations, decelerations, and cover greater distances at high speeds during a match. This suggests that left-side players assume a more active and physically demanding role, requiring specific training programs to enhance anaerobic performance and recovery strategies [31]. Additionally, left-side players tend to execute more cross-court strokes and winning shots, while right-side players perform more parallel strokes and lobs. The presence of a left-handed player on the right side can alter tactical behaviors, enabling both players to cover the center of the court with their dominant hand, which increases offensive efficiency [32].

Likewise, gender differences in playing characteristics have been explored, showing different technical-tactical profiles, movement patterns and physical responses during matches according to sex [33–36].

Therefore, the aim of the present study was to examine fitness parameters and to compare the differences regarding the side of play during matches in both men’s and women’s padel.

## MATERIALS AND METHODS

Based on the hypothesis that padel players engage in short, highintensity actions involving frequent directional changes, overhead strokes, and jumping actions, an observational and descriptive study was designed to investigate differences in physical capabilities according to playing position (left- or right-side players) in professional male and female players. This analysis was conducted using data collected during a national gathering of padel players, employing a series of standardized anthropometric and physical performance tests.

### Participants

This study involved a total of thirty professional padel players (male: n = 15; female: n = 15) ranked within the top 50 of the World Padel Tour (WPT) rankings, were distributed in two groups: players playing on the right side (men n = 7; women n = 7) and the players playing on the left side (men n = 8; women n = 8). A power analysis was conducted using G*Power 3.1 software (University of Düsseldorf, Düsseldorf, Germany). The analysis indicated that a sample size of 15 participants would yield a statistical power of 0.60, assuming an alpha level of 0.05 and an effect size of 0.5 for a paired t-test. All players were right-handed, except for one male player who was left-handed. Table 1 provides a detailed summary of the participants’ profiles (anthropometric data and training information). Prior to participation, players were thoroughly informed about the study procedures, including any potential risks, and provided written informed consent. The research protocol was approved by the Clinical Research Ethics Committee of the Department of Health and Consumption of the Government of Aragon, Spain (21/2012).

**TABLE 1:** Differences between participants’ characteristics to side of play, divided by gender

	Male padel player (n = 15)				Female padel player (n = 15)			

	Left side player (n = 8) (SD)	Right side player (n = 7) (SD)	ES	p	Left side player (n = 8) (SD)	Right side player (n = 7) (SD)	ES	p
Experience (years)	9.00 (3.25)	7.14 (2.73)	0.62	0.257	11.22 (4.68)	10.42 (1.27)	0.27	0.672
Age (years)	30.25 (7.57)	24.14 (4.29)	1.03	0.083	29.22 (4.87)	31.14 (3.24)	0.47	0.385
Weight (kg)	81.60 (9.71)	74.41 (5.31)	0.96	0.106	59.21 (5.40)	60.89 (3.84)	0.36	0.499
Height (cm)	180.03 (3.31)	175.55 (3.37)	1.34	0.022*	166.90 (5.31)	166.30 (4.49)	0.12	0.814
Fat Mass (%)	15.33 (5.55)	11.45 (3.02)	0.91	0.125	20.56 (3.81)	18.40 (7.13)	0.39	0.494

Note: SD = Standard Deviation; ES = Effect Size; p = significant differences;

* , p value p < 0.05.

### Procedure

The evaluation protocol was carried out over four non-consecutive days, ensuring a minimum recovery period of 72 hours between sessions. The sequence of assessments was as follows: on the first day, isometric handgrip strength (HD), sit-and-reach (SaR) flexibility, and the 10 × 5 shuttle test were performed; the second day included countermovement (CMJ), squat (SJ) and Abalakov (ABK) jump tests; the third day focused on the one-repetition maximum test (1-RM); and the final day involved anthropometric measurements and *maximum oxygen uptake* (VO_2max_) evaluation. Two researchers well versed in and experienced with the protocol carry out the evaluations. In order to avoid bias in the performance evaluations, the researchers were unaware of the position in which the participants played. To maintain consistency, all tests were administered in the same sequence, using standardized equipment, measurement protocols, and experienced operators. The assessments took place in a controlled laboratory environment, with a temperature of 21 °C and relative humidity ranging from 50% to 60%. Participants were familiarized with the tests before the evaluation began. A dynamic warm-up routine was carried out 15 min before the tests, which included joint mobilization, dynamic stretching, progressive hopping drills, and vertical jumps of increasing intensity. Participants were instructed to avoid caffeine and other stimulants, as well as any intense physical activity, for at least 48 hours prior to testing. No injuries ofrincapacities were reported.

### Measurements

*Anthropometry:* Anthropometric data were obtained from all participants, including height, measured using a fixed stadiometer with an accuracy of ± 0.1 cm (Seca 220, Hamburg, Germany), and weight, recorded with a digital scale offering an accuracy of ± 0.1 kg (Seca 714, Hamburg, Germany).

*Fat Mass (FM):* Anthropometric assessments were conducted by a single experienced evaluator utilizing specialized equipment: a skinfold calliper (Holtain Ltd., Crymych, UK) with a precision of 0.2 mm and a flexible anthropometric steel tape (Holtain Ltd., Crymych, UK) accurate to 0.1 cm. Measurements included eight skinfold sites (biceps brachii, triceps, subscapular, iliac crest, supraspinal, abdominal, thigh, and medial calf), four girths (relaxed and tensed biceps brachii, thigh, and calf), and three bone widths (biepicondylar femur, biepicondylar humerus, and bistyloid wrist). Body Mass Index (BMI) was calculated from body weight (kg) divided by height squared (m^2^), and body fat percentage was estimated using a body density formula [37], followed by a body fat percentage calculation [38].

VO_2max_: It was determined through a graded treadmill test in a controlled laboratory setting on a Pulsar HP treadmill (Cosmos, Nussdorf, Germany). After a 3-min warm-up at 6 km · h^−1^, the test began at a 1% incline, with an initial speed of 8 km · h^−1^, increasing by 1 km · h^−1^ per minute until the subject reached exhaustion. Expired gases were collected and analyzed using an Oxycon Pro gas analyzer (Jaegger, Germany).

*1-RM Test:* Maximal strength, or 1-RM [39], was assessed based on established guidelines for strength analysis in untrained individuals. The 1-RM was indirectly calculated using a predictive formula based on repetitions to fatigue [39], applied across various exercises: bench press, leg extension, squat, leg curl, leg press, lat pulldown, overhead press, and shoulder press. Each test began with two preliminary series—one for technique familiarization and a warm-up set at approximately 30–50% of perceived 1-RM—allowing multiple repetitions with one-min rest intervals. Participants made two to three attempts with load increments of 10–20%, with rest intervals of 3–5-min. Testing concluded when players achieved five or fewer repetitions in each exercise. A single evaluator supervised all 1-RM tests to ensure consistent execution.

*CMJ test:* Lower limb reactive strength was measured with a twolegged vertical CMJ without arm swing, following a standardized protocol [40] on a contact mat (Newtest Powertimer 300-series, Newtest Oy, Finland). The correct knee angle of 90° was verified by an investigator. Each participant performed two maximal-effort jumps, with 45-s of passive rest between attempts. The highest jump height (in cm) was recorded, based on flight time.

*SJ test:* Leg power performance was evaluated using a SJ from a static, semi-squatting position (90°) on a contact mat (Newtest Powertimer 300-series, Newtest Oy, Finland), following an established protocol [40]. Participants performed two maximal efforts with 45-s rest intervals. The highest jump height (in cm), calculated from flight time, was recorded.

*ABK test:* A CMJ with arm swing was executed on a contact mat (Newtest Powertimer 300-series, Newtest Oy, Finland). Each participant performed two maximum-effort attempts with a 45-s rest between jumps, and the highest jump height (in cm) was recorded based on flight time.

*HD test:* HD was measured in both hands. Players extended their testing arm vertically without body contact, using a portable hand dynamometer (Smedley III T-18A, Takei, Tokyo, Japan) with a measurement range from 0 to 100 kg in 0.5 kg increments and ± 2 kg accuracy. Each participant performed two maximal 5-s isometric contractions with each hand, with a 2-min rest between trials. The dynamometer was adjusted for each participant’s hand size, and the highest strength measurement from each hand was used in data analysis.

*SaR Test:* Flexibility of the lower back and hamstrings was assessed with the traditional SaR test. Participants sat with legs fully extended and the soles of their feet flat against a box. With palms down and hands either stacked or side-by-side, they reached forward along a measurement line, maintaining the stretch for 1–2-s. Distance reached (in cm) was recorded.

*10 × 5 m Shuttle Test:* Speed and agility, including acceleration and deceleration abilities, were assessed using a 10 × 5 m shuttle run. Time was recorded using a photocell gate system (Newtest Powertimer 300-series, Newtest Oy, Finland) and accompanying software (Newtest PowertimerPC 2.0 for Windows), with an accuracy of ± 0.001 s. Timing began and ended as players crossed the gate.

*Take-Off Reaction Test (Newtest*^®^ – *Finland):* This test evaluated participants’ response to a lateral red light, indicating a required movement direction (left or right) from a ready position [41]. The subject sprinted laterally from a contact mat to a photocell positioned 5 m away. Each participant completed 12 attempts (six to each side), and the best result was recorded. Measured parameters were reaction speed reaction time (RSRT), reaction speed acceleration time (RSAT), and reaction speed total time (RSTT). RSRT refers to the time elapsed between the appearance of the stimulus (the red light) and the initial movement of the subject. Reaction Speed Acceleration Time (RSAT) measures the time taken by the subject to reach their maximum speed after initiating the movement. RSTT is the sum of RSRT and RSAT, representing the total time from the stimulus onset until the subject reaches the photocells. Anticipation was considered when reaction time was below 150 ms, and photocells were positioned at hip height to avoid interference from arm movement.

### Statistical Analysis

Data were presented as mean (standard deviation). Descriptive statistics, including mean and standard deviation, outlier analysis, and Shapiro–Wilk tests for normality assessment, were performed. Levene’s test confirmed variance homogeneity. Student’s t-test assessed variable differences based on playing side. Due to the sample size, Hedges’ g was used to calculate effect size [42], with interpretations as trivial (< 0.25), low (0.25–0.50), moderate (0.50–1.0), and high (> 1) [43]. A significance level of p < .05 was applied, and analyses were conducted using SPSS (version 25.0; SPSS, Inc., Chicago, IL, USA).

### RESULTS

Those male players who played on the left side showed a statistically significant difference in height (ES: 1.34; p = 0.022) from those who played on the right side. Despite not showing a significant difference, experience (ES: 0.62), age (ES: 1.03), weight (ES: 0.96), and FM (ES: 0.91) showed a moderate-high effect size. On the other hand, no differences were observed between female players according to their side of the game (Table 1).

According to strength performance (Table 2), moderate differences were observed in RM S (ES: 0.83), RM LC (ES: 0.53) in male players who played on the left side, compared to those on the right side, in both cases without significant differences. In contrast, high differences in CMJ (ES: 1.21; p = 0.037), SJ (ES: 0.85; p = 0.005) were shown in players who played on the right side, when compared to those on the left side. Also, moderate tendency in ABK (ES: 0.84), were observed, though in this case it was not significant (Table 2). On the other hand, moderate differences were found in RM LP (ES: 0.63), RM S (ES: 0.68) and HD D (ES: 0.69) in female players who played on the left side, while moderate differences were shown in HD ND (ES: 0.60) in players who played on the right side, also not significant.

**TABLE 2:** Differences between participants’ strength performance to side of play, divided by gender

	Male padel player				Female padel player			

	Left side player (n = 8)	Right side player (n = 7) (SD)	ES	p	Left side player (n = 8) (SD)	Right side player (n = 7) (SD)	ES	p
RM BP (kg)	61.93 (27.31)	72.37 (22.26)	0.42	0.460	38.19 (8.73)	37.97 (11.76)	0.02	0.966
RM LP (kg)	134.34 (47.27)	149.13 (19.74)	0.44	0.506	93.44 (16.31)	83.34 (15.88)	0.63	0.284
RM LE (kg)	138.41 (33.77)	146.84 (41.88)	0.22	0.673	88.47 (17.50)	79.78 (23.74)	0.42	0.430
RM S (kg)	205.81 (80.70)	148.84 (55.81)	0.83	0.141	145.62 (26.83)	125.11 (33.62)	0.68	0.231
RM LC (kg)	143.14 (76.01)	118.79 (15.39)	0.53	0.422	75.94 (24.51)	69.25 (16.32)	0.33	0.551
RM LaP (kg)	88.35 (18.52)	89.81 (13.00)	0.09	0.867	46.58 (5.29)	44.15 (5.70)	0.44	0.408
RM O (kg)P	61.3 (10.88)	60.28 (17.09)	0.07	0.892	25.05 (6.25)	22.10 (9.18)	0.38	0.496
RM SP (kg)	101.12 (51.79)	109.97 (57.00)	0.16	0.775	38.54 (8.53)	35.11 (10.23)	0.37	0.491
HD D (kg)	50.58 (10.72)	51.77 (7.86)	0.13	0.814	35.37 (5.70)	31.60 (5.18)	0.69	0.194
HD ND (kg)	46.07 (10.12)	46.32 (5.64)	0.03	0.954	28.28 (3.12)	26.30 (3.50)	0.60	0.252
CMJ (cm)	28.7 (6.59)	37.14 (7.36)	1.21	0.037*	24.60 (5.33)	24.03 (5.89)	0.10	0.847
SJ (cm)	21.20 (3.70)	32.85 (8.91)	1.85	0.005*	22.33 (5.13)	20.66 (4.25)	0.36	0.509
ABK (cm)	35.51 (7.08)	43.14 (11.13)	0.84	0.132	28.15 (5.96)	30.60 (4.63)	0.46	0.396

Note: SD = Standard Deviation; ES = Effect Size; RM = one-repetition maximum; HD = Hand dynamometry with dominant and non-dominant hand; BP = bench press; LE = leg extension; S = Squat; LC = leg curl; LP = leg press; LaP = lat pulldowns; OP = overhead press; SP = shoulder press; CMJ = countermovement jump; SJ = squat jump; ABK = Abalakov jump;

* , p value p < 0.05

Comparing endurance, flexibility and speed performance in male players, those who played in the left side showed no significant differences but obtained moderate bias in 10 × 5 (ES: 0.80), RSTT L (ES: 0.87), RSTT R (ES: 0.94), RSRT _R (ES: 0.93) (Table 3). In women, players who played in the left side obtained a trend of better results (ES: 0.58), while players from the left side performed better results in SaR (ES: 0.53) RSAT L (ES: 0.58) RSRT R (ES: 0.55), also without significant differences (Table 3).

**TABLE 3:** Differences between participants’ endurance, flexibility and speed performance according to side of play, divided by gender

	Male padel player				Female padel player			

	Left side player (n = 8) (SD)	Right side player (n = 7) (SD)	ES	p	Left side player (n = 8) (SD)	Right side player (n = 7) (SD)	ES	p
VO_2max_ (mL/kg/min)	54.16 (3.76)	56.87 (9.72)	0.40	0.478	48.09 (4.70)	45.46 (4.36)	0.58	0.300
SaR (cm)	29.11 (11.42)	27.31 (9.89)	0.17	0.751	32.46 (3.98)	36.19 (10.04)	0.53	0.379
10 × 5 (s)	16.58 (1.24)	15.68 (1.02)	0.80	0.152	17.64 (1.06)	17.59 (1.31)	0.04	0.935
RSTT L (s)	2.05 (0.13)	1.95 (0.10)	0.87	0.102	2.14 (0.08)	2.20 (0.19)	0.44	0.442
RSTT R (s)	1.99 (0.09)	1.91 (0.08)	0.94	0.131	2.15 (0.10)	2.20 (.17)	0.37	0.526
RSRT L (s)	0.77 (0.23)	0.70 (0.10)	0.42	0.461	0.61 (0.20)	0.57 (.17)	0.22	0.685
RSRT R (s)	0.81 (0.09)	0.68 (0.19)	0.93	0.127	0.62 (0.13)	0.68 (0.09)	0.55	0.336
RSAT L (s)	1.28 (0.16)	1.24 (0.17)	0.24	0.686	1.52 (0.18)	1.63 (0.20)	0.58	0.336
RSAT R (s)	1.18 (0.07)	1.23 (0.21)	0.36	0.541	1.54 (.16)	1.52 (0.21)	0.11	0.915


Note: SD = Standard Deviation; ES = Effect Size; SaR: Sit and reach, RSTT: Reaction speed total time in the left (L) or right (R) displacement, RSRT: Reaction speed reaction time in the left or right displacement; RSAT Reaction speed acceleration time in the left or right displacement.

### DISCUSSION

Padel players perform different actions depending on their side of play, which implies different physical demands; therefore, physical preparation should consider this aspect. Consequently, the aim of the present study was to explore fitness parameters and to compare the differences based on the side of play during matches in both men’s and women’s padel. The main findings of this study were: 1) male padel players on the left side showed better results in age, weight, height, FM, RM S, RM LC, ABK compared to those on the right side. 2) male padel players on the right side showed better results in 10 × 5, RSTT L, RSTT R and RSRT R compared to those on the left side. 3) Female padel players on the left side demonstrated moderately better performance in RM LP, RM S, HD D, HD ND, VO_2_max, RSRT R, and RSAT L compared to those on the right side.

Male padel players presented slightly higher weight values (81.6 ± 9.71 kg left-side players and 74.41 ± 5.31 kg right-side players) than in other padel studies, where the results are 78.52 ± 8.65 kg [44] or 79.1 ± 9.7 kg [2]. In turn, the results showed a moderate difference (ES: 0.96) between players playing on the left side compared to players playing on the right side, being the former slightly higher. This may be attributed to the role of the left-side padel player, particularly regarding the distances covered and their participation in the active phases of the game [29, 30]. These values were higher than those shown in other sports such as badminton (between 75.1 kg and 64.8 kg) [45–48], where the demands of the game may explain this difference [49].

Similar results to the previously mentioned were found for height. The results obtained in the present study, male players (180.03 ± 3.31 left-side players and 175.55 ± 3.37 cm right-side players), showed similar values to previous research (177.1 to 177.4 cm) [2, 44, 50]. Male padel players also presented high (ES: 1.34) and significant differences (p = 0.02), with higher values obtained by those playing on the left side. This difference may be related to the technicaltactical differences that players have depending on their side of play [51]. In both men and women, the results obtained are similar to those obtained in previous racket sports [46–48, 52].

The FM obtained from male padel players were higher in players playing on the left side (15.33%) than in right-side players (11.45%), resulting in a moderate difference (ES: 0.91). Compared to other studies, this data was slightly lower (21.3 ± 5.6%) [2]. Given that the various studies reported different levels of play across age groups, it is difficult to determine the cause of these results, except for the possibility of an improvement in the sample’s physical condition.

Moderate differences could be seen in the isometric strength of hand pressure in both HD D (ES: 0.69) and HD ND (ES: 0.60) in left-handed female padel players compared to right-handed female padel players. The results obtained were slightly higher than those shown in another investigation (right hand 26.0 kg and left hand 23.8 kg) [24]. However, this previous study differentiated limbs without taking dominance into consideration, so it is difficult to make a comparison between them. Nevertheless, the results obtained in the present study indicate that hand pressure is important in padel, as they are in line with previous research on different racket sports [2, 53]. Although the effect size is moderate to large, it is important to note that the p-value exceeds 0.05, indicating that the results are not statistically significant.

One of the main findings of this investigation was the high and significant difference obtained in the CMJ (ES: 1.21 and p = 0.03) and SJ (ES: 1.85; p < 0.01) vertical jump tests in male padel players, with better results for those playing on the right side. However, left-side male players showed moderate outcomes (ES: 0.84) in ABK compared to right-side male players. These differences in results depending on the test and side of play could be due to the physical demands [29, 30], as well as the technical-tactical differences of each player depending on their side of play [51], which may be interesting to take into consideration when planning training sessions. Furthermore, these results highlighted the need for athlete development in jumping power compared to other racket sports, such as table tennis [54], although not to the same extent as in badminton [55, 56].

VO_2max_ values for left-side female padel players (48.09 ± 4.70 mL/kg/min) were higher than those for right-side players (45.46 ± 4.36 mL/kg/min), showing moderate differences. These results are slightly higher than those found in previous research [24]. Nevertheless, this data suggests that VO_2max_ is an important factor in padel, although not a limiting one [57], without the relevance it may have in other racket sports such as tennis [58]. Statistical significance was not achieved (p > 0.05), despite the presence of a moderate to large effect size in the findings.

For the assessment of maximum strength, the 1-RM Test was performed in various movements. The results obtained in the present study were slightly superior to other racket sports, such as badminton (143.2 ± 17.3 kg) [55] or table tennis (86.77 ± 5.24 kg) [54]. This may be due to the rules of padel, where the dimensions of the court and the use of walls [19] may require a higher demand of muscular endurance during the game. One of the main findings was the moderate improvements in RM S (ES: 0.83) and RM LC (ES: 0.53) in male padel players playing on the left side over the right. Thus, the following results suggested that, as a result of the technical-tactical demands of the different roles of padel players [51], players on the left side obtained better results in maximum strength in order to maintain a good strength base for the subsequent execution of power in different offensive strokes, while players on the right side obtained better results in jumping, given the explosiveness of defensive return actions against attacking strokes. In turn, in female padel players, moderate results in RM S (ES: 0.68) were reported for those players on the left side over those on the right side. These results were consistent with those of the male padel players in the present study, supporting the previously mentioned suggestion regarding the strength demands depending on the side of play. The lack of statistical significance (p > 0.05) contrasts with the moderate to large effect size observed in the analysis.

Regarding the shuttle test, male padel players who played on the right side obtained moderately better results in agility (ES: 0.81) than the players on the left side. These results were in accordance to those obtained in the strength demonstrations, since the players on the right side, due to their game demands, require more explosive actions and agility for the correct execution of defensive strokes. However, due to the limited literature in which agility tests differ across studies [2, 25], it is difficult to compare the current results with those previously found.

Several studies have analysed the influence of reaction speed and effective inhibitory control in racket sports, and their relationship with sport performance [41, 59, 60]. In the present study, it could be observed how male padel players showed moderately better results in RSTT L (ES: 0.87), RSTT R (ES: 0.94) and RSRT R (ES: 0.93). In the case of female padel players, the moderate difference in RSRT R (ES: 0.55) and RSAT L (ES: 0.58) is noteworthy. A moderate to large effect size was detected, but the p-value, being greater than 0.05, precludes any claims of statistical significance. These results were similar to previous findings [41, 59], suggesting that there are no differences between different racket sports, so future studies are warranted.

There were some limitations to the present study. The first limitation was the scarcity of information in padel regarding various physical conditioning parameters based on the side of play and gender, which made comparisons difficult. At the same time, limited information has been found on other technical-tactical aspects of the game in each sex, so it cannot be related to the physical parameters of the athletes. Additionally, the study did not take into account the playing style of the players, which could significantly influence in physical outcomes. Therefore, future studies are warranted.

### CONCLUSIONS

In male players, those who played on the left side exhibited greater muscle mass and strength levels, measured as 1-RM, in specific exercises such as the RM S and RM LC. Conversely, players on the right side achieved superior outcomes in vertical jump assessments, including the CMJ and SJ. Among female players, moderate differences were found in VO_2max_ and HD (both, dominant and non-dominant hands), with higher values observed in left-side players.

## Practical applications

The findings highlight the need for tailored physical preparation in padel based on the side of play and gender. For male left-side players, strength training and aerobic conditioning are recommended to support offensive play and endurance. Right-side male players should focus on explosive power and agility drills to enhance defensive actions. Female left-side players benefit from lower-body strength training and VO_2max_ improvement, while right-side female players should prioritize agility and reaction speed exercises. These recommendations can help coaches design individualized programs to optimize performance.
